# Supplementary material for: Multi-Gene Mutation Profiling by Targeted Next-Generation Sequencing in Premenopausal Breast Cancer
Source: Genes (Basel). 2022 Jul 29;13(8):1362. doi: 10.3390/genes13081362 (PMC9407588; doi:10.3390/genes13081362)
Supplement: Supplementary file 1 [file genes-13-01362-s001.zip › genes-1823096-supplementary.pdf]

Supplementary Table S1: Germline and somatic mutations of 54 premenopausal women with breast cancer as determined by NGS in relation to each patient's distinct clinicopathological characteristics.

Patient ID Number	Germline Pathogenic mutations	Germline VUS mutations	Somatic pathogenic mutations	Somatic VUS mutations	Age (at Initial Diagnosis)	Stage	Breast Location	T	N	M	Histological Type	ER (at Initial Diagnosis)	PR (at Initial Diagnosis)	HER2 (at Initial Diagnosis)	KI67 (at Initial Diagnosis)	Grade	Molecular Subtype
2	(-)	(-)	(-)	(-)	28	III A	LEFT	2	2	0	IDC	POSITIVE	POSITIVE	POSITIVE	≥20%	3	LUMINAL B (HER2 POSITIVE)
3	(-)	(-)	TP53 (c.424_433del, p. Pro142Cysfs*25)	(-)	36	IA	RIGHT	1	0	0	IDC	POSITIVE	POSITIVE	POSITIVE	<20%	2	LUMINAL B (HER2 POSITIVE)
4	(-)	(-)	N/A	N/A	32	IIA	LEFT	2	0	0	IDC	POSITIVE	POSITIVE	NEGATIVE	<20%	3	LUMINAL A
5	(-)	(-)	PIK3CA (c.3140A>G, p. His1047Arg)<br>PIK3CA (c.1090G>A, p. Gly364Arg)	BRCA1 (c.3743C>T, p.a1248v)<br>TP53 (c.622G>A, p.d208n)	38	IIB	RIGHT	2	1	0	IDC	POSITIVE	POSITIVE	NEGATIVE	≥20%	3	LUMINAL B (HER2 NEGATIVE)
6	(-)	(-)	(-)	(-)	40	IIIC	LEFT	3	3	0	ILC	NEGATIVE	NEGATIVE	POSITIVE	<20%	3	HER2 ENRICHED
7	(-)	(-)	TP53 (c.743G>A, p. Arg248Gln)	KMT2C (c.4873G>A, p. Glu1625Lys)	37	III A	LEFT	3	2	0	IDC	POSITIVE	POSITIVE	NEGATIVE	≥20%	3	LUMINAL B (HER2 NEGATIVE)
8	TP53 (c824g>a, p. Cys275Tyr)	(-)	TP53 (c.824g>a, p. Cys255Tyr)	ROS1 (c433A>C, p. Thr145Pro)<br>RET (c.1684A>T, p. Thr562Ser)	32	III A	LEFT	4	x	0	IDC	NEGATIVE	NEGATIVE	POSITIVE	≥20%	3	HER2 ENRICHED
9	(-)	(-)	TP53 (c.990del, p. Gln331Argfs*14)	MET (c.4090C>T, p. Pro1364Ser)<br>NF1 (c.563C>A, p. Ala188Glu)	40	III A	RIGHT	3	1	0	IDC	NEGATIVE	POSITIVE	NEGATIVE	≥20%	3	LUMINAL B (HER2 NEGATIVE)
10	(-)	(-)	N/A	N/A	40	IIIC	RIGHT	x	x	0	IDC	NEGATIVE	NEGATIVE	POSITIVE	≥20%	2	HER2 ENRICHED
11	(-)	(-)	N/A	N/A	37	IIB	RIGHT	2	1	0	IDC	NEGATIVE	NEGATIVE	NEGATIVE	≥20%	3	TNBC

12	(-)	(-)	PTEN (c.99_100ins, p. Ala34fs)	FGFR3 (c.2272G>A, p. Asp758Asn)<br>ROS1 (c.1135C>G, p. Gln379Glu)<br>BRAF (c.1159G>A, p. Gly387Arg)<br>TP53 (c.691del, p. Thr231ProfsTer16)	40	IIIA	LEFT	3	1	0	IDC	POSITIVE	POSITIVE	NEGATIVE	<20%	2	LUMINAL A
13	BRCA1 (c.5266dup, p. Gln1756Profs*74)<br>MUTYH (c.452A>G, p. Tyr151Cys)	(-)	(-)	(-)	40	IIIC	LEFT	2	3	0	IDC	NEGATIVE	NEGATIVE	NEGATIVE	≥20%	3	TNBC
14	CHECK2 (c.1232G>A, p. Trp411*)	(-)	N/A	N/A	40	IIIA	RIGHT	N/A	N/A	N/A	IDC	POSITIVE	POSITIVE	NEGATIVE	≥20%	3	LUMINAL B (HER2 NEGATIVE)
15	(-)	(-)	ATM (c.494T>G, p.Leu165*)	ATM (c.482A>C, p. Gln161Pro)	47	IA	RIGHT	1	0	0	IDC	NEGATIVE	NEGATIVE	NEGATIVE	≥20%	3	TNBC
16	(-)	(-)	AKT1 (c.49G>A, p.Glu17Lys)	PTEN (c.481A>G, p. Arg161Gly)<br>RAD50 (c.443A>G, p. Lys148Arg)	52	IIB	RIGHT AND LEFT	R:T2, L:T2	R:N0, L:N1	0	ILC	POSITIVE	POSITIVE	NEGATIVE	<20%	3	LUMINAL A
17	(-)	(-)	N/A	N/A	47	IIA	LEFT	1	1	0	IDC	POSITIVE	POSITIVE	NEGATIVE	<20%	2	LUMINAL A
18	(-)	(-)	N/A	N/A	44	IIIA	LEFT	1	2	0	IDC	POSITIVE	POSITIVE	NEGATIVE	≥20%	2	LUMINAL B (HER2 NEGATIVE)
19	(-)	(-)	N/A	N/A	42	IIB	LEFT	3	0	0	IDC	POSITIVE	POSITIVE	NEGATIVE	<20%	2	LUMINAL A
20	(-)	(-)	N/A	N/A	45	IA	RIGHT	1	0	0	IDC	NEGATIVE	NEGATIVE	NEGATIVE	≥20%	3	TNBC
21	(-)	(-)	N/A	N/A	44	IA	RIGHT	1	0	0	IDC	POSITIVE	POSITIVE	POSITIVE	≥20%	3	LUMINAL B

																	(HER2 POSITIVE)
22	(-)	(-)	N/A	N/A	46	IIA	LEFT	2	0	0	IDC	POSITIVE	POSITIVE	POSITIVE	≥20%	3	LUMINAL B (HER2 POSITIVE)
23	(-)	(-)	N/A	N/A	48	IIIB	LEFT	4	2	0	IDC	POSITIVE	POSITIVE	NEGATIVE	≥20%	3	LUMINAL B (HER2 NEGATIVE)
24	(-)	(-)	N/A	N/A	43	IIB	LEFT	2	1	0	IDC	POSITIVE	POSITIVE	NEGATIVE	<20%	2	LUMINAL A
25	(-)	(-)	TP53 (c.824G>A, p. Cys275Tyr)	KMT2C (c.7826G>A, p. Arg2609Gln)<br>RB1 (c.1988A>G, p. Asn663Ser)<br>NOTCH1 (c.2453T>C, p. Leu818Pro)	45	IA	LEFT	1	0	0	IDC	NEGATIVE	NEGATIVE	POSITIVE	≥20%	3	HER2 ENRICHED
26	(-)	(-)	N/A	N/A	46	IA	RIGHT	1	0	0	IDC	POSITIVE	POSITIVE	NEGATIVE	<20%	1	LUMINAL A
27	(-)	(-)	PIK3CA (c.3140A>G, p. His1047Arg)<br>TP53 (c.743G>A, p. Arg248Gln)	ROS1 (c.500G>A, p. Arg167Gln)	52	IV	LEFT	x	x	1	IDC	POSITIVE	POSITIVE	NEGATIVE	≥20%	3	LUMINAL B (HER2 NEGATIVE)
28	(-)	ATM (c.7475T>G, p. Leu2492Arg)	N/A	N/A	33	IIIA	RIGHT	3	1	0	IDC	POSITIVE	POSITIVE	NEGATIVE	<20%	3	LUMINAL A
29	(-)	BRCA1 (c.3649T>C, p. Ser1217Pro)	(-)	(-)	20	IIIC	LEFT	2	3	0	IDC	POSITIVE	POSITIVE	NEGATIVE	≥20%	3	LUMINAL B (HER2 NEGATIVE)
30	(-)	BRCA2 (c. 1342C>T, p. Arg448Cys)	N/A	N/A	35	IA	LEFT	1	0	0	IDC	POSITIVE	POSITIVE	NEGATIVE	≥20%	2	LUMINAL B (HER2 NEGATIVE)
31	(-)	BRCA2 (c. 9613_9614delinsCT, p. Ala3205Leu)	(-)	BRCA2 (c. 9613_9614delinsCT, p. Ala3205Leu)	34	IV	RIGHT	x	x	1	IDC	POSITIVE	POSITIVE	NEGATIVE	≥20%	2	LUMINAL B (HER2

		RAD51D (c.412A>C, p. Asn138His)															NEGATIVE)
32	BRCA1 (c.3700_3704del, p. Val1234Glnfs*8)<br>CHECK2 (c.470T>C, p. Ile157Thr)	BRCA2 (c.352C>T, p. Arg118Cys)	N/A	N/A	37	IIB	RIGHT	2	1	0	IDC	POSITIVE	NEGATIVE	NEGATIVE	≥20%	3	LUMINAL B (HER2 NEGATIVE)
33	(-)	BRCA2 (c.9867T>G, p. Phe3289Leu)	TP53 (c.85_86del, p. Asn29GlnfsTer13)	BRCA2 (c.9867T>G, p. Phe3289Leu)<br>ROS1 (c.433A>C, p. Thr145Pro)	46	IIIC	LEFT	1	3	0	IDC	POSITIVE	POSITIVE	POSITIVE	≥20%	3	LUMINAL B (HER2 POSITIVE)
34	(-)	BRCA2 (c.8386C>T, p. Pro2796Ser)	N/A	N/A	38	IA	RIGHT	1	0	0	IDC	POSITIVE	POSITIVE	NEGATIVE	≥20%	3	LUMINAL B (HER2 NEGATIVE)
35	(-)	BRIP1 (c. 2285G>A, p. Arg762His)	N/A	N/A	33	III A	RIGHT	3	2	0	IDC	POSITIVE	POSITIVE	NEGATIVE	≥20%	3	LUMINAL B (HER2 NEGATIVE)
36	(-)	CHECK2 (c.480A>G, p. Ile160Met)	(-)	(-)	29	IIIC	LEFT	4	3	0	IDC	POSITIVE	NEGATIVE	NEGATIVE	≥20%	2	LUMINAL B (HER2 NEGATIVE)
37	(-)	CHECK2 (c.190G>A, p. Glu64Lys)	(-)	FGFR1 (c.2192_*del, p. Lys731_Arg732fs*159)	43	IIA	RIGHT	2	0	0	IDC	POSITIVE	NEGATIVE	POSITIVE	≥20%	3	LUMINAL B (HER2 POSITIVE)
38	(-)	CHECK2 (c. 1175C>T, p. Ala392Val)	N/A	N/A	37	IV	RIGHT	2	1	1	IDC	POSITIVE	POSITIVE	NEGATIVE	≥20%	3	LUMINAL B (HER2 NEGATIVE)
39	(-)	CHECK2 (c.470T>C, Ile157Thr)	(-)	(-)	40	IIA	LEFT	2	0	0	IDC	POSITIVE	POSITIVE	POSITIVE	≥20%	2	LUMINAL B (HER2 POSITIVE)

40	N/A	N/A	(-)	(-)	31	IA	RIGHT	1	0	0	IDC	NEGATIVE	NEGATIVE	NEGATIVE	≥20%	3	TNBC
41	N/A	N/A	PI3KCA (c.1624G>A, p.Glu542Lys)	NF1 (c.7301T>C, p.Leu2434Pro)	39	IIA	LEFT	1	1	0	IDC	POSITIVE	POSITIVE	POSITIVE	≥20%	3	LUMINAL B (HER2 POSITIVE)
42	N/A	N/A	(-)	(-)	33	IIB	RIGHT	2	1	0	IDC	POSITIVE	POSITIVE	NEGATIVE	≥20%	3	LUMINAL B (HER2 NEGATIVE)
43	N/A	N/A	PIK3CA (c.3140A>G, p. His1047Arg)	RB1 (c.43G>A, p. Ala15Thr)	30	IIIC	LEFT	2	3	0	IDC	POSITIVE	POSITIVE	NEGATIVE	<20%	3	LUMINAL A
44	N/A	N/A	TP53 (c.488A>G, p. Tyr163Cys)	RAD50 (c.1094G>A, p. Arg365Gln)<br>TP53 (c.562_564delInsAA, p. Leu188Lysfs*59)<br>CCND1 (c.724-2A>C)	37	IIB	RIGHT	2	1	0	IDC	POSITIVE	POSITIVE	POSITIVE	≥20%	3	LUMINAL B (HER2 POSITIVE)
45	N/A	N/A	PIK3CA (c.3140A>T, p. His1047Leu)	TP53 (c.815T>C, p. Val272Ala) IDH1 (c.949C>T, p.R317C)<br>CTNNB1 (c.1206del, p. Thr404Leufs*11)<br>KMT2C (c.3521A>G, p. Gln1174Arg)	38	IIA	LEFT	1	1	0	IDC	POSITIVE	POSITIVE	NEGATIVE	<20%	3	LUMINAL A
46	N/A	N/A	TP53 (c.586C>T, p.Arg196*)	(-)	40	IIA	RIGHT	2	0	0	IDC	NEGATIVE	NEGATIVE	NEGATIVE	≥20%	3	TNBC
47	N/A	N/A	(-)	(-)	44	IIA	RIGHT	2	0	0	IDC	POSITIVE	POSITIVE	NEGATIVE	<20%	2	LUMINAL A
48	N/A	N/A	(-)	ROS1 (c.433A>C, p. Thr145Pro)<br>NOTCH1 (c.7487_7489del, p. Asn2496del)	45	IV	LEFT	x	x	1	IDC	POSITIVE	POSITIVE	POSITIVE	N/A	3	LUMINAL B (HER2 POSITIVE)

49	N/A	N/A	PIK3CA (exon2: c.115G>A, p.Glu39Lys) TP53 (c.818G>T, p.Arg273Leu)	(-)	49	IA	Left	1	0	0	IDC	POSITIVE	POSITIVE	POSITIVE	<20%	3	LUMINAL B (HER2 POSITIVE)
50	N/A	N/A	PIK3CA (c.1624G>A, p. Glu542Lys)	RAD50 (c.2604T>G, p. Asn868Lys)	50	IA	RIGHT	1	0	0	IDC	POSITIVE	POSITIVE	NEGATIVE	<20%	1	LUMINAL A
51	N/A	N/A	PIK3CA (exon21: c.3140A>G, p. His1047Arg)<br>ERBB2 (exon19: c.2264T>C, p. Leu755Ser)	RAD50 (c.980G>A, p. Arg327His)<br>TP53 (c.304A>T, p. Thr102Ser)<br>NF1 (c.2573C>G, p. Ser858Cys)	47	IA	LEFT+RIGHT	L:1a R: 1c	L:0, R:0	0	IDC	POSITIVE	POSITIVE	NEGATIVE	≥20%	2	LUMINAL B (HER2 NEGATIVE)
52	N/A	N/A	BRCA2 (c.5576_5579del, p. Ile1859LysfsTer3 TTAA)	(-)	50	III A	LEFT	1	2	0	IDC	POSITIVE	POSITIVE	NEGATIVE	<20%	2	LUMINAL A
53	(-)	PMS2 (c.1999G>A, p. Glu667Lys)	(-)	(-)	44	III A	LEFT	1	2	0	IDC	POSITIVE	POSITIVE	NEGATIVE	≥20%	3	LUMINAL B (HER2 NEGATIVE)
54	(-)	RAD51C (c.80T>C, p. Leu27Pro)	N/A	N/A	48	IA	LEFT	1	0	0	IDC	NEGATIVE	NEGATIVE	NEGATIVE	≥20%	3	TNBC
55	N/A	N/A	PIK3CA (c.1633G>A, p. Glu545Lys),<br>PTEN (c.923delG, p. Arg308LeufsTer9)<br>PTEN (c.923delA, p. Asp310IleufsTer7)	(-)	39	IV	LEFT+ RIGHT	L:1, R:3	L:2, R:3	1	`IDC	POSITIVE	POSITIVE	NEGATIVE	≥20%	2	LUMINAL B (HER2 NEGATIVE)
